# Chronic Fatigue Syndrome: Diagnosis, Treatment, and Future Direction

**DOI:** 10.7759/cureus.70616

**Published:** 2024-10-01

**Authors:** B. Sue Graves, Mitsu Patel, Hailey Newgent, Gauri Parvathy, Ahmad Nasri, Jillene Moxam, Gurnoor S Gill, Vivek Sawhney, Manish Gupta

**Affiliations:** 1 Exercise Science and Health Promotion, Florida Atlantic University, Boca Raton, USA; 2 Medicine, Smt. Nathiba Hargovandas Lakhmichand Municipal Medical College, Ahmedabad, IND; 3 Occupational Therapy, University of Florida, Jacksonville, USA; 4 Medicine, Tbilisi State Medical University, Tbilisi, GEO; 5 Technology and Clinical Trials, Advanced Research, Deerfield Beach, USA; 6 Orthopaedics, University of Florida College of Medicine, Jacksonville, USA; 7 Charles E. Schmidt College of Medicine, Florida Atlantic University, Boca Raton, USA

**Keywords:** center for disease control, chronic fatigue, chronic fatigue syndrome, general fatigue, international consensus criteria

## Abstract

Myalgic encephalomyelitis (ME), also known as chronic fatigue syndrome (CFS), is a complex, chronic condition marked by persistent, debilitating fatigue that is not alleviated by rest and often worsens with physical or mental exertion. Along with fatigue, patients experience various symptoms, including cognitive impairments, post-exertional malaise, muscle and joint pain, sleep disturbances, and immune system dysfunction. Diagnosing CFS/ME is challenging due to the absence of definitive biomarkers, the overlap of symptoms with other conditions, and the lack of standardized diagnostic criteria. This comprehensive literature review aims to contribute to the understanding of CFS/ME, including its diagnosis, pathophysiology, differential diagnosis, treatment, and future directions.

## Introduction and background

Myalgic encephalomyelitis (ME), also known as chronic fatigue syndrome (CFS), is characterized by severe, persistent exhaustion that significantly limits daily activities and is not relieved by rest. This fatigue often worsens with physical or mental exertion and is accompanied by other debilitating symptoms. In addition to fatigue, individuals may experience a range of symptoms, such as post-exertional malaise, neuro-cognitive symptoms (confusion or slow thinking), muscle pain, joint pain, sleep disturbances, hypersensitivity to stimuli, flu-like symptoms, autonomic dysfunction (weakness and tremors), and immune system abnormalities that are usually made worse by exertion and not better explained by a different underlying etiology [1]. CFS/ME is a severe illness that can lead to significant physical impairment, leaving patients immobile and requiring extensive care. This care may include hospitalizations for acute episodes, home support for daily activities, and outpatient management involving medications, physical therapy, and mental health services [2]. A multidisciplinary team of healthcare professionals, home health aides, and family caregivers often provides this care [2]. Neuro-cognitive deficits, chronic exhaustion, and flu-like symptoms are some of the disease's hallmarks [3]. An epidemiological investigation found that about half of CFS/ME patients are unable to work [4]. According to prior research, 48% of patients cannot engage in any constructive activity at their worst, and 87% cannot work [3]. Following modest physical, orthostatic, and cognitive exertion, the malaise and its associated symptoms deteriorate markedly, known as post-exertional malaise (PEM) [5]. This primary symptom often appears 24 hours after overexertion and causes a considerable drop in the patient's energy and activity level (at least 50%) [6]. Other symptoms include problems with memory and attention, commonly referred to as "brain fog,” slowed information processing, difficulty articulating ideas or finding the correct words, inability to multitask or sustain concentration on activities, short-term memory impairment, and a shorter attention span [7]. Allergies or sensitivity to any kind of stimulus, including light, noise, odors, extreme temperature, chemicals, and simple conversations, are frequent patient complaints [8]. Patients with severe illness may express an inability to communicate, lie in bed in the dark, take frequent showers, and the inability to watch TV or listen to music [2]. Depression, anxiety, and decreased motivation or interest in previously favored activities are also very common symptoms [2].

Compared to the general population, those with CFS/ME also have a higher risk of suicide [9]. Multiple risk factors, including social and cultural elements such as unsupportive interactions, social isolation, and negative attributions, affect people with CFS/ME. Suicidal thoughts may arise from the condition's effect on jobs and everyday activities, which may result in feelings of inadequacy and diminished self-worth [10]. Another important risk factor for suicide is chronic pain. Furthermore, patient's perceptions of their condition may change because of unidentified comorbidities, thereby impacting suicidal thoughts [10].

Between 836,000 and 2.5 million Americans have been estimated to have CFS/ME, as noted in the 2015 IOM report [11]. Among the cases identified, less than 20% reported receiving a CFS/ME diagnosis from a healthcare provider [12]. Among persons diagnosed with CFS/ME, the ratio of women to men can be as high as four to one [12]. CFS/ME is less common in children than in adults but is more prevalent in adolescents than in younger children [13]. Diagnosed mostly in people between 40 and 60 years old. CFS/ME occurs in all ethnic and racial groups [14]. Factors that may increase the risk of developing CFS/ME include age, sex, and medical comorbidities [15]. Young to middle-aged adults and women are most affected. Patients with a history of other complex medical conditions, such as fibromyalgia or postural orthostatic tachycardia syndrome, are also at risk.

The COVID-19 pandemic has brought significant attention to post-viral fatigue syndromes, particularly due to its association with conditions resembling CFS/ME [16]. However, the absence of COVID-19 in the introduction and background of this review is notable, given that ongoing research increasingly links the virus to the development of CFS/ME-like symptoms. Often referred to as "long COVID" or "post-acute sequelae of SARS-CoV-2 infection" (PASC), this post-infection phenomenon shares many similarities with CFS/ME, including persistent, debilitating fatigue, post-exertional malaise, cognitive difficulties ("brain fog"), muscle and joint pain, sleep disturbances, and autonomic dysfunction [16,17]. Studies indicate that up to 10-30% of individuals infected with SARS-CoV-2 experience long-term symptoms consistent with CFS/ME, regardless of the severity of the initial illness. This high prevalence points to COVID-19's potential as a trigger for CFS/ME, similar to other viral infections such as Epstein-Barr virus, which have historically been associated with CFS/ME onset [18].

The mechanisms by which COVID-19 may lead to a CFS/ME-like condition are an area of active investigation. One of the proposed mechanisms is immune dysregulation; COVID-19 is known to provoke a robust inflammatory response, including a cytokine storm, which may lead to persistent immune activation even after the acute infection has resolved [16]. This ongoing inflammation could contribute to chronic fatigue and other symptoms characteristic of CFS/ME [17]. Autonomic nervous system dysfunction is another potential link between COVID-19 and CFS/ME. Many long COVID patients exhibit symptoms such as orthostatic intolerance, irregular heart rate, and blood pressure abnormalities, which mirror autonomic dysfunction observed in CFS/ME [18]. Additionally, there is evidence suggesting mitochondrial dysfunction in long COVID, aligning with similar findings in CFS/ME where impaired mitochondrial energy production may underlie the profound fatigue and reduced exercise tolerance [19].

Another hypothesis centers on viral persistence, suggesting that fragments of the SARS-CoV-2 virus may remain in the body, continuously triggering an immune response and leading to prolonged symptoms [20]. This theory is akin to those proposed for other infections that can precede CFS/ME. Furthermore, SARS-CoV-2 has been shown to affect the central nervous system, potentially causing neuroinflammation. This could explain neurological symptoms like cognitive dysfunction and "brain fog," which are hallmark features of both long COVID and CFS/ME [20].

The diagnosis of CFS/ME is challenging due to the absence of definitive biomarkers, the overlap of symptoms with other conditions, and a lack of standardized diagnostic criteria. The aim of this comprehensive literature review is to contribute to the understanding of CFS/ME, including its diagnosis, pathophysiology, differential diagnosis, treatment, and future directions.

The paper conducted a detailed search of databases such as PubMed, Cochrane Library, and MEDLINE using keywords such as "chronic fatigue syndrome," "myalgic encephalomyelitis," "graded exercise therapy," "cognitive behavioral therapy," and "pharmacological treatments." Inclusion criteria focused on peer-reviewed, English-language studies related to adult ME/CFS patients, addressing diagnosis, treatment, etiology, and pathophysiology.

This narrative review summarized data from the International Consensus Criteria (ICC) and the Centers for Disease Control (CDC) to highlight their importance. This article aims to contribute to understanding CFS/ME, encompassing its diagnosis, treatment, and potential underlying mechanisms. The etiology and pathophysiology were reviewed, emphasizing immune system malfunction and deficiencies in energy production. Several alternative diagnosis and evaluation instruments for CFS/ME were included. Treatment modalities were discussed, including medication and non-pharmacological therapies, such as graded exercise therapy (GET) and cognitive behavioral therapy (CBT). This narrative review offers valuable insights for healthcare practitioners and researchers to optimize the management of this challenging condition while identifying areas for further research and improvement.

## Review

Diagnosis

Currently, no diagnostic test or validated biomarker exists for CFS/ME. However, certain results provide a sense of hope for succeeding diagnostic biomarkers or the use of an immune signature assay [21]. Because of the diagnostic challenge in identifying CFS/ME, individuals may experience their ailment for years before an official diagnostic confirmation.

The Center for Disease Control (CDC) has set standards to identify CFS/ME [14] (Table 1).

**Table 1:** Center for Disease Control (CDC) criteria for chronic fatigue syndrome

Category	Symptoms
Primary Symptoms	Clinically evaluated, unexplained, persistent, or relapsing chronic fatigue that is: new or definite onset (has not been lifelong), not the result of ongoing exertion, not substantially alleviated by rest, lasting for at least six months, and a substantial reduction in previous levels of occupational, educational, social, or personal activities.
Additional Symptoms	At least four of the following symptoms must occur simultaneously: memory or concentration problems, sore throat, tender lymph nodes, muscle pain, joint pain that does not swell or turn red, headaches that are new in pattern or severity, restless sleep, and post-exertional malaise (PEM) that lasts more than 24 hours.

The International Consensus Criteria (ICC) for diagnosis of CFS/ME include post-exertional neuroimmune exhaustion (PENE), defined as weariness exacerbated by mental, emotional, or physical strain requiring a protracted time of recuperation [12] and at least one symptom across three categories. This includes specific symptoms of neurological impairment, immune, gastrointestinal, and genitourinary impairment, and energy metabolism and transport impairments (Table 2).

**Table 2:** International Consensus Criteria (ICC) for the diagnosis of CFS/ME Ref. [21]

Category	Requirement	Symptoms
Post-exertional Neuroimmune Exhaustion (PENE)	Compulsory	Marked, rapid physical and/or cognitive fatigability in response to exertion. Exacerbation of symptoms like flu-like feelings, pain, and worsening of other symptoms. Exhaustion may occur immediately or after a delay, lasting days, weeks, or longer.
Neurological impairments	1 or more symptoms from 3 of 4 categories	1) Neurocognitive impairments: Slowed thought, impaired concentration, memory issues, slowed speech, and exertional dyslexia. 2) Pain: Chronic headaches with muscle tension. 3) Sleep disturbance: Insomnia, unrefreshing sleep. 4) Motor issues: Weakness.
Immune, GI, and GU impairments	1 or more symptoms from 3 of 5 categories	1) Flu-like symptoms. 2) Susceptibility to viral infections. 3) GI symptoms: Nausea, bloating, irritable bowel syndrome. 4) GU symptoms: Urine urgency, nocturia. 5) Sensitivities to food, odors, or chemicals.
Energy production/transportation impairments	1 or more symptoms	1) Cardiovascular: Orthostatic intolerance, palpitations, and vertigo. 2) Respiratory: Labored breathing, air hunger, chest wall muscle tiredness. 3) Loss of thermostatic stability. 4) Intolerance to temperature extremes.

MFI assesses different dimensions of fatigue, including general fatigue, physical fatigue, reduced activity, reduced motivation, and mental fatigue [14]. It helps evaluate the impact of fatigue on various aspects of life [14]. The 36-Item Short Form Survey (SF-36) is a generic health survey that assesses multiple aspects of health-related quality of life, including physical functioning, social functioning, vitality, mental health, and pain. The DePaul Symptom Questionnaire (DSQ) is a comprehensive self-report tool that assesses a wide range of symptoms associated with CFS/ME, allowing for a detailed evaluation of the illness [22]. The PROMIS Fatigue Short Form measures the impact of fatigue on physical, mental, and social well-being, providing a broader assessment of fatigue-related quality of life [23]. The Chalder fatigue scale is commonly used to assess the severity of fatigue in various medical conditions, including CFS/ME [18]. Functional MRI is a non-invasive technique that measures brain activity by detecting changes in blood flow. It has been used to study cerebral blood flow and brain connectivity in individuals with CFS/ME to explore differences in brain function and assess cognitive impairments [24]. Single-photon emission computed tomography (SPECT) is a nuclear medicine imaging technique using radioactive tracers to evaluate blood flow in organs, including the brain. This procedure is used in CFS/ME research to study cerebral blood flow and identify potential abnormalities. PET is another nuclear medicine imaging method that uses radioactive tracers to assess metabolic activity in tissues. A study has used PET scans to explore brain metabolism and function in individuals with CFS/ME. Lastly, magnetic resonance spectroscopy (MRS) is an MRI-based technique that measures the chemical composition of tissues. It has been utilized in CFS/ME research to study metabolite levels in the brain and muscle tissue [24,25] (Table 1).

The Fukuda Criteria is one of the most established and widely used diagnostic tools for CFS, also known as ME. Developed by the CDC in 1994, the criteria aimed to provide a standardized and practical approach for identifying CFS/ME in both clinical and research settings [26]. This was especially crucial at the time, given the diverse and often non-specific symptoms of CFS/ME, which made it challenging for clinicians to diagnose the condition consistently. By offering a defined set of guidelines, the Fukuda Criteria has played a significant role in shaping our understanding and approach to CFS/ME (Table 3).

**Table 3:** Fukuda Criteria for CFS/ME: Symptom requirements

Criteria	Symptoms
Exhibit at least 4 or more of the following 8 symptoms for at least 6 consecutive months	1) impaired memory or concentration; 2) sore throat without active infection; 3) tender lymph nodes, particularly in the neck or armpits; 4) widespread muscle pain; 5) multi-joint pain without swelling or redness; 6) new, severe headaches; 7) unrefreshing sleep; and 8) post-exertional malaise lasting over 24 hours, marked by a worsening of symptoms after physical or mental exertion

While the Fukuda Criteria has been a cornerstone for CFS/ME diagnosis for decades, it has faced several criticisms and limitations. One of the most significant points of contention is that the criteria do not require post-exertional malaise (PEM) as a mandatory symptom [27]. PEM is often considered a hallmark of CFS/ME, involving a profound exacerbation of symptoms following physical or cognitive exertion that can last for days or weeks [27]. By not making PEM a compulsory part of the diagnostic criteria, the Fukuda guidelines may overlook a key aspect of the condition, potentially leading to misdiagnosis or inclusion of patients with other fatigue-related disorders [27].

Another limitation of the Fukuda Criteria is its breadth, which can encompass a wide range of conditions presenting with fatigue. This has raised concerns about diagnostic inconsistencies, as some patients meeting the Fukuda Criteria may not truly have CFS/ME but rather other medical or psychological conditions that manifest similar symptoms [26]. The criteria also do not account for some of the multisystem dysfunctions observed in CFS/ME, such as orthostatic intolerance, neurocognitive impairments, autonomic nervous system disturbances, and immune dysregulation [28]. These features are increasingly recognized in newer diagnostic criteria, such as the International Consensus Criteria (ICC) and the Institute of Medicine (IOM) criteria, which place a stronger emphasis on symptoms such as PEM, autonomic dysfunction, and neurological manifestations. By not including these elements, the Fukuda Criteria may miss the full spectrum of CFS/ME's impact on patients [27].

Furthermore, the subjectivity of symptom assessment under the Fukuda Criteria poses challenges for both clinicians and researchers [27]. Many symptoms, such as fatigue, pain, and cognitive difficulties, are self-reported and lack objective measures, making it difficult to quantify severity or track changes over time [28]. This subjectivity has led to variability in how different practitioners and studies interpret and apply the criteria, potentially affecting the consistency of diagnosis and research outcomes. Additionally, the Fukuda Criteria do not address the fluctuating nature of CFS/ME symptoms, where patients may experience periods of relative improvement and worsening [28]. This episodic pattern complicates the diagnosis, as patients may not always meet the criteria at a given point in time, even though they have the condition.

Despite these limitations, the Fukuda Criteria remain an important tool, particularly in research settings where a standard definition is needed to identify study populations. Its widespread adoption over the past few decades has facilitated a large body of research into CFS/ME, contributing to our understanding of the condition's epidemiology, risk factors, and potential pathophysiological mechanisms [27]. However, there is a growing consensus in the medical community that updates to the diagnostic criteria are necessary to more accurately reflect the complexities of CFS/ME. This includes integrating recent findings on immune system involvement, autonomic dysfunction, and metabolic abnormalities, as well as emphasizing the significance of post-exertional malaise as a core symptom [29]. In this context, the Fukuda Criteria serve as both a foundation and a stepping stone towards more comprehensive and precise diagnostic guidelines [26].

Etiology and pathophysiology

The exact cause of CFS/ME remains unclear; however, in recent years, various studies have provided preliminary evidence suggesting a multifaceted etiology involving neuro-immune-endocrine interactions, metabolic changes, and genetic factors [3].

Dysfunctional immune mechanisms, including altered CD8+ T-cells, reduced T regulatory cells, and NK cell activity, may play a role [28]. Some patients show damage-associated molecular patterns (DAMPs) and abnormal cytokine levels, suggesting an association with inflammation [28]. CFS/ME is often triggered by infections, including Epstein-Barr virus (EBV), which can lead to immune system dysregulation. EBV may cause an abnormal, persistent immune response characterized by the overproduction of pro-inflammatory cytokines, altered CD8+ T-cell function, and reduced T regulatory cell activity, contributing to chronic inflammation [30,31]. Additionally, EBV's ability to remain latent in B-cells may result in a continuous, low-level immune response, potentially driving the prolonged symptoms seen in CFS/ME [32]. Although EBV is a key suspect, its exact role in the development of CFS/ME remains unclear, highlighting the need for further research [32].

CFS/ME may also have autoimmune components, and the gut's involvement is under investigation. In fact, recent research suggests that the gut plays a significant role in CFS/ME pathophysiology through mechanisms involving gut microbiome dysbiosis, increased gut permeability, immune modulation, disrupted energy metabolism, and the gut-brain axis [33]. Patients with CFS/ME often exhibit gut dysbiosis, characterized by reduced levels of beneficial bacteria such as Bifidobacterium and Lactobacillus and an overgrowth of pro-inflammatory bacteria, contributing to systemic inflammation. This dysbiosis may lead to a "leaky gut," where the compromised gut barrier allows harmful substances, such as lipopolysaccharides (LPS), to enter the bloodstream, triggering an immune response and promoting inflammation [33]. These immune changes, including impaired regulatory T-cell function, may explain the autoimmune-like symptoms observed in CFS/ME. Moreover, the altered gut microbiome composition affects the production of short-chain fatty acids (SCFAs) such as butyrate, which are crucial for maintaining gut barrier integrity, modulating immune function, and supporting energy metabolism. Reduced SCFA production in CFS/ME patients may contribute to mitochondrial dysfunction and the condition's hallmark fatigue [33]. The gut-brain axis is also implicated, as dysbiosis and leaky gut can alter neurotransmitter production and cause neuroinflammation, potentially resulting in cognitive dysfunction or "brain fog." Given these connections, interventions such as probiotics, prebiotics, dietary changes, and fecal microbiota transplantation (FMT) are being explored to restore gut health and mitigate symptoms, though further research is needed to clarify these mechanisms and develop effective treatments [33].

Gender differences exist, with more women affected, possibly due to hormonal influences and immune responses [6]. Estrogen, the primary female sex hormone, is known to have significant effects on immune function. In women, higher levels of estrogen tend to promote a more robust immune response, which may contribute to the heightened risk of autoimmune conditions and immune dysregulation observed in CFS/ME [34]. This heightened immune activity can result in an overproduction of pro-inflammatory cytokines and altered T-cell function, both of which have been implicated in the pathophysiology of CFS/ME [34]. Additionally, hormonal fluctuations during different life stages - such as puberty, menstruation, pregnancy, and menopause - may influence the onset or exacerbation of CFS/ME symptoms. For instance, fluctuations in estrogen and progesterone levels during the menstrual cycle are thought to impact the immune system and may worsen symptoms such as fatigue, pain, and cognitive dysfunction in CFS/ME patients. Pregnancy, with its associated changes in immune regulation and hormone levels, has also been noted to trigger or alter the course of CFS/ME [34]. The post-menopausal period, marked by a significant decrease in estrogen levels, may further contribute to immune dysregulation and the persistence or worsening of symptoms. Female reproductive events might trigger or affect CFS/ME [34]. Currently, valid diagnostic markers and a clear understanding of the disease's mechanisms remain challenging.

CFS/ME is also associated with impaired energy generation, including reduced mitochondrial function and oxidative stress [28]. Studies have found higher lactate levels, reactive oxygen species (ROS), and oxidative stress markers in CFS/ME patients [5]. There are abnormalities in glycolysis and amino acid metabolism, leading to inefficient energy production and post-exertional malaise [35,36]. Some researchers suggested that gut fermentation products such as propionate may contribute to oxidative stress and mitochondrial dysfunction.

MRI scans have revealed structural and functional brain abnormalities in CFS/ME patients, primarily affecting areas related to executive functions, cognition, memory, and perception, though these abnormalities occur across various regions [37]. One study found reduced connectivity in the brainstem and hippocampus, particularly in the reticular activating system (RAS) [37]. The severity of these deficits was found to correlate with the patient's symptoms and disease progression, suggesting central nervous system involvement, which may help explain the distinctive fatigue experienced by CFS/ME patients. However, direct pathological evidence of brain inflammation in CFS/ME remains limited.

CFS/ME appears to have a genetic component, supported by higher concordance rates in identical twins and the increased prevalence of certain gene variants related to autoimmunity in post-infectious CFS/ME patients. Specific single nucleotide polymorphisms (SNPs), such as PTPN22 rs2476601 and CTLA4 rs3087243, are associated with infection-triggered CFS/ME, while other SNPs, such as IRF5 rs3807306 and TNF rs1799724, have decreased allele frequencies in CFS/ME patients who do not have infection-triggered onset [38]. These genetic variants may contribute to impaired mucosal immunity, potentially increasing susceptibility to CFS/ME following infection. However, more research is necessary to fully elucidate the genetic factors involved.

Differential diagnosis

When diagnosing CFS/ME, healthcare providers face the challenge of distinguishing it from a variety of other conditions that present with similar symptoms. CFS/ME is known to significantly impact instrumental activities of daily living (IADLs), such as cleaning, driving, and managing finances. The symptoms overlap with conditions such as chronic fatigue, rheumatological disorders (e.g., fibromyalgia, systemic lupus erythematosus), psychiatric disorders (e.g., depression, bipolar disorder), Lyme disease, multiple sclerosis, and sleep apnea, making differential diagnosis particularly complex​ (Table 4). The table clarifies these differences by outlining the key features of these disorders, highlighting that while fatigue is a common symptom, CFS/ME uniquely involves a multisystem impact, including post-exertional malaise, unrefreshing sleep, and cognitive impairment. Accurate diagnosis is further complicated by the absence of definitive laboratory markers, the need to rule out other possible causes, and the subjective nature of many symptoms. This differential diagnosis process requires a comprehensive medical history, clinical examination, and specific diagnostic testing where applicable to avoid misdiagnosis and to initiate effective treatment strategies.

**Table 4:** Differential diagnosis of chronic fatigue

Disorder Category	Key Features
Chronic Fatigue	Chronic fatigue is a symptom rather than a specific disorder and can result from various underlying conditions such as sleep disorders, depression, anemia, or thyroid dysfunction. Unlike CFS/ME, chronic fatigue does not include the hallmark symptoms of post-exertional malaise, unrefreshing sleep, or cognitive impairment. Chronic fatigue is generally less severe and lacks the characteristic multisystem involvement seen in CFS/ME. Diagnostic evaluation for chronic fatigue involves identifying and addressing the underlying causes, which may not require the same comprehensive, multifactorial approach used for CFS/ME [3].
Rheumatological Disorders	These disorders include fibromyalgia, polymyalgia rheumatica, polymyositis, systemic lupus erythematosus (SLE), and rheumatoid arthritis. Key features involve persistent musculoskeletal pain, joint stiffness, and, in some cases, inflammation. Fibromyalgia is characterized by widespread pain and tender points without joint swelling, whereas SLE and rheumatoid arthritis often present with joint swelling, skin rashes, and specific autoantibodies (e.g., ANA in lupus, rheumatoid factor in rheumatoid arthritis). Unlike CFS/ME, these conditions may show positive laboratory findings, such as elevated inflammatory markers (ESR, CRP) or autoantibodies, which aid in their diagnosis [37].
Psychiatric Disorders	Disorders including major depressive disorder, anxiety, bipolar disorder, and somatoform disorders, can present with fatigue, sleep disturbances, and cognitive impairments similar to CFS/ME. However, in psychiatric conditions, fatigue often occurs alongside other hallmark features, such as persistent feelings of sadness, loss of interest, mood swings, or anxiety symptoms. Unlike CFS/ME, post-exertional malaise is not a defining characteristic, and cognitive dysfunction tends to be more related to mood disturbances rather than an underlying physical illness. Accurate diagnosis relies on a thorough mental health history and assessment to differentiate these disorders from CFS/ME.
Lyme Disease	Lyme disease, caused by the Borrelia burgdorferi bacterium transmitted via tick bites, can mimic CFS/ME symptoms such as fatigue, joint pain, and cognitive difficulties. However, Lyme disease typically presents with a characteristic erythema migrans (bull's-eye) rash and may involve neurological symptoms like facial palsy. Diagnosis is supported by a history of tick exposure and confirmed through serologic testing for Lyme-specific antibodies. This differentiation is crucial, as Lyme disease requires specific antibiotic treatment, unlike the management strategies for CFS/ME [6].
Multiple Sclerosis(MS)	Multiple Sclerosis (MS) is an autoimmune disorder that affects the central nervous system, leading to symptoms like fatigue, cognitive dysfunction, and muscle weakness. Unlike CFS/ME, MS often presents with additional neurological signs, such as visual disturbances, coordination problems, and sensory deficits. Diagnostic tools such as magnetic resonance imaging (MRI) showing demyelination in the central nervous system and specific markers (e.g., oligoclonal bands in cerebrospinal fluid) help distinguish MS from CFS/ME. The table emphasizes these distinguishing features, aiding in differential diagnosis [6].
Neurological Disorders	Fatigue is a primary symptom of multiple sclerosis. Dementia, characterized primarily by cognitive impairment, can create diagnostic challenges, as can conditions like pseudodementia [4]. Neurological conditions such as dementia, Parkinson’s disease, and epilepsy can also exhibit fatigue, cognitive impairment, and motor dysfunction similar to CFS/ME. However, these conditions usually have other distinct neurological features. For instance, dementia primarily presents with progressive memory loss and executive function decline, while Parkinson’s involves motor symptoms like tremors and rigidity. Diagnostic imaging, neuropsychological testing, and the identification of specific neurological signs are critical in differentiating these disorders from CFS/ME.
Respiratory Disorders	Chronic respiratory disorders, including chronic obstructive pulmonary disease (COPD) and sarcoidosis, can cause chronic fatigue and sleep disturbances. However, respiratory symptoms such as shortness of breath, chronic cough, wheezing, and oxygen desaturation are more characteristic of these conditions than CFS/ME. Diagnosis is supported by pulmonary function tests for COPD or polysomnography for OSA, differentiating them from CFS/ME, where respiratory issues are not primary features [3].
Sleep Apnea	Undiagnosed obstructive sleep apnea can manifest as fatigue and unrefreshing sleep, which are key diagnostic criteria for chronic fatigue syndrome. Diagnosis is confirmed through polysomnography [3].

Pharmacological Management

Only a limited number of randomized controlled trials (RCTs) have investigated pharmacological treatments for CFS/ME [39,40]. Although there is no cure for the condition, various medications are used to alleviate and manage its symptoms. Symptom control is particularly crucial in cases requiring personalized treatment plans. The range of medications prescribed for CFS/ME is broad, including over-the-counter options such as pain relievers and non-steroidal anti-inflammatory drugs (NSAIDs), along with anticonvulsants, antidepressants, narcotics, antivirals, and immunomodulatory drugs (Table 5) [40].

**Table 5:** Pharmacological management of chronic fatigue

Medication class	Examples	Purpose
NSAIDS	Ibuprofen, Naproxen	Relieve frequent or severe joint and muscle pain, headaches, and fevers and also reduce inflammation [3].
Anticonvulsants	Gabapentin, pregabalin	These medications are often prescribed for pain and sleep issues [39] and tend to be most effective when used to treat nerve pain.
Antidepressants	Antidepressant medications: A) Selective serotonin reuptake inhibitors, B) Tricyclic Antidepressants	Antidepressants, often prescribed for CFS/ME due to their link with depression, show varying success. SSRIs like fluoxetine, sertraline, and paroxetine can help with chronic pain, fibromyalgia, anxiety, and mood disorders [12]. Tricyclics, such as amitriptyline, relieve insomnia and low energy, while others (doxepin, desipramine, nortriptyline, clomipramine, imipramine) improve sleep and reduce pain, typically requiring 3-4 weeks for symptom relief [40].
Narcotics	Tramadol, codeine, morphine	Narcotics are prescribed for pain that does not respond to over-the-counter medications [39]. They are typically reserved for the most severe cases due to the risk of addiction and are used only for short-term management.
Antiviral and Immunomodulatory Drugs	Ritatolimod (Ampligen), acyclovir, valganciclovir	Although gaining FDA approval has been challenging, ritatolimod (Ampligen) shows promise as an antiviral and immunomodulatory treatment for CFS/ME [41]. Nucleotide analogs like acyclovir and valganciclovir may offer benefits, but they come with risks, including potential complications such as renal failure [42].
Interferons	Not well established due to limited high-quality trials	The effectiveness of interferons in treating CFS/ME remains uncertain, as there is a lack of high-quality trials to support their use [1].
Steroids	Hydrocortisone, fludrocortisone, hydrocortisone plus fludrocortisone	Steroid treatments, including cortisol and thyroid hormones, have also been explored for CFS/ME [40]. Seven randomized controlled trials (RCTs) have been conducted: four involving hydrocortisone, two with fludrocortisone, and one trial combining hydrocortisone and fludrocortisone [1].

Nonpharmacological Management of Chronic Fatigue

Nonpharmacological treatment approaches include cognitive behavior therapy (CBT), GET, and adaptive pacing therapy (APT) (Table 6). GET and APT are two distinct approaches to managing CFS/ME. GET is a structured treatment that involves a gradual increase in physical activity, based on the idea that deconditioning contributes to CFS/ME symptoms. It requires patients to follow a tailored exercise plan, which slowly increases in intensity over time. However, this approach is controversial, as many patients report a worsening of symptoms, particularly post-exertional malaise, a key characteristic of CFS/ME. In contrast, APT focuses on helping patients manage their energy levels by balancing activity with rest. It emphasizes listening to the body and setting realistic activity levels to avoid overexertion. Rather than aiming to increase physical activity, APT adapts to the patient’s current limitations, aiming to prevent symptom flare-ups. These differing approaches lead to significantly different outcomes in CFS/ME management.

**Table 6:** Nonpharmacological management of chronic fatigue

Treatment Approach	Description
Cognitive Behavior Therapy (CBT) and Graded Exercise therapy (GET)	CBT and GET have been shown to offer no significant improvement in quality of life, reduction in the number of CFS symptoms, or measurable objective progress, and they negatively impact work and benefit status. As a result, CBT and GET should not be recommended or prescribed for ME/CFS patients [43]. Ongoing research may help enhance the effectiveness of these treatments in the future.
Adaptive Pacing Therapy	APT aims to adjust patient behavior by considering symptom fluctuations and delayed recovery from exercise [44]. It helps set realistic exercise goals and balances rest to avoid overexertion. When combined with GET, pacing demonstrated significantly better outcomes than relaxation and flexibility treatments [44]. APT appears to be a more effective, safer, and more acceptable approach for managing CFS/ME, making it a recommended option

Controversies and Debates

The controversies and debates surrounding CFS/ME have significant implications for both patient care and future research. The psychological versus biological causes debate, for instance, directly influences treatment approaches and funding allocation for research. If CFS/ME is primarily viewed as a psychological condition, interventions such as CBT and GET might be prioritized, potentially overlooking the need for biomedical research and treatments targeting immune, endocrine, or neurological dysfunctions [45]. On the other hand, considering CFS/ME as a biological illness opens up avenues for exploring treatments such as antiviral medications, immune-modulating therapies, and strategies addressing mitochondrial dysfunction [45].

The post-viral or chronic infection hypothesis has gained traction, especially with the emergence of conditions such as long COVID. If viral infections, such as EBV or human herpesvirus 6 (HHV-6), are identified as key triggers for CFS/ME, this could lead to a focus on antiviral treatments, vaccines, and early intervention strategies in patients with acute viral illnesses to potentially prevent the development of CFS/ME [20]. Similarly, the autoimmune hypothesis highlights the possibility that CFS/ME could involve an inappropriate immune response, pointing to immunotherapies or treatments aimed at restoring immune system balance as potential interventions.

By discussing these debates, the section underscores the complexity of CFS/ME and the need for a multifaceted research approach. Acknowledging the different hypotheses not only justifies varied treatment approaches but also emphasizes the urgency for more robust research to resolve these debates. Ultimately, the controversies shape the direction of clinical practice, research funding, and public health policies, making them essential to include for a comprehensive understanding of CFS/ME.

Psychological vs. Biological Causes

One of the longstanding controversies surrounding CFS/ME is the debate over whether it is primarily a psychological or biological condition [46]. Some researchers and healthcare professionals have suggested that psychological factors, such as stress or somatization, play a significant role in the development of CFS/ME. On the other hand, others argue that compelling evidence exists pointing to underlying biological abnormalities, including immune dysfunction, neuroendocrine imbalances, and viral infections [46]. The debate continues to shape how the condition is perceived and treated [46].

Post-viral or Chronic Infection Hypothesis

Some researchers and patients believe that CFS/ME may be triggered or perpetuated by a viral infection. EBV and HHV-6 are among the viruses frequently implicated. While evidence of viral involvement has been found in some CFS/ME cases, the exact relationship between viral infections and CFS/ME remains uncertain [3].

Autoimmune Hypothesis

Another debated theory is that CFS/ME may have an autoimmune component. Some researchers have proposed that the immune system may target and attack the body's own tissues, leading to symptoms characteristic of CFS/ME. However, the evidence supporting an autoimmune etiology for CFS/ME is still inconclusive [18,41].

GET and Pacing Controversy

The use of GET for CFS/ME has been a subject of controversy. While some studies suggest potential benefits, patient experiences vary widely, with some reporting improvement and others experiencing worsened symptoms.

The quality of evidence regarding GET for CFS/ME is a contentious issue [47]. Many studies supporting GET have been criticized for methodological flaws, including small sample sizes, lack of appropriate control groups, and potential biases in patient selection [47,48]. Additionally, some studies rely on subjective outcome measures, such as self-reported improvement in symptoms, which can introduce bias and limit the reliability of findings. Moreover, there are concerns about patient adherence to GET protocols. Due to the nature of CFS/ME, patients may experience fluctuating levels of fatigue and post-exertional malaise, making it difficult for them to adhere to a structured exercise regimen [47,48]. This variability in adherence further complicates the interpretation of study results, as it becomes challenging to differentiate between the effects of the therapy and the natural course of the illness.

Critics also point out that many studies do not adequately address the potential harms of GET, such as exacerbation of symptoms [47,48]. As a result, the controversy surrounding GET is fueled not only by conflicting study outcomes but also by limitations in the design and execution of these studies. Therefore, more rigorous, patient-centered research is needed to provide clearer guidance on the use of GET in CFS/ME management [47,48].

Patient Perspectives and Challenges

Due to the absence of diagnostic indicators and symptom overlap with other disorders, CFS/ME patients face a variety of patient experiences and obstacles, including difficulty getting a quick diagnosis. Getting the right treatment from specialists might be difficult, which can be frustrating. Feelings of invalidation and loneliness may result from stigma and misunderstandings regarding CFS/ME. The effects of CFS/ME on the body and mind might interfere with everyday living and social interactions.

Future directions

While genetic factors are believed to play a role in the development of CFS/ME, specific genetic markers and their functional significance remain largely unknown. Future research should identify genetic variations associated with CFS/ME susceptibility and disease severity to shed light on underlying biological mechanisms [37]. Future research should also seek to identify reliable and specific biomarkers that can aid in diagnosis, subtyping, and monitoring treatment responses, as there are currently no definitive biomarkers for diagnosing CFS/ME or monitoring disease progression. In fact, the progression of CFS/ME is highly variable; some patients remain stable, while others experience worsening symptoms triggered by physical exertion, stress, or infections. Although full recovery is rare, partial improvements occur in some individuals. Rather than a linear progression, CFS/ME involves symptom fluctuations and potential long-term disability. Future research is needed to identify biomarkers that track these changes, providing insights into symptom management and targeted interventions.

Biomarkers could include immune markers, neuroendocrine indicators, or metabolic profiles [12]. Additionally, identifying distinct subtypes of CFS/ME based on specific clinical and biological characteristics is an essential research area, as it could lead to more targeted and personalized treatments. The gut microbiome's role in CFS/ME is also an emerging area of interest. Understanding the interactions between the gut microbiota and the immune system and how they may contribute to CFS/ME pathogenesis could pave the way for novel therapeutic approaches. Lastly, investigating the role of environmental triggers, such as infections, toxins, or stressors, in the development and exacerbation of CFS/ME could provide insights into prevention and targeted interventions.

Advances in genomics have enabled researchers to study the genetic basis of CFS/ME more comprehensively [37]. Genome-wide association studies (GWAS) and transcriptomic analyses can help identify genetic variants and gene expression patterns associated with CFS/ME, providing insights into underlying molecular mechanisms [37]. Metabolomics allows for the systematic analysis of small molecules (metabolites) in biological samples, representing another potential for technological advances. This technology can help identify metabolic alterations and dysregulations associated with CFS/ME, potentially leading to the discovery of disease-specific metabolic profiles or biomarkers [16]. Proteomics represents another potential as it enables the study of the entire complement of proteins in a biological sample. By examining protein expression patterns and post-translational modifications, proteomic analyses can provide information on molecular pathways involved in CFS/ME pathogenesis [30]. Advanced neuroimaging techniques, such as functional MRI (fMRI) and diffusion tensor imaging (DTI), can provide insights into brain structure and function in individuals with CFS/ME [39]. These technologies can help elucidate the neurological basis of CFS/ME and identify potential biomarkers related to brain abnormalities. Additionally, integrating large-scale omics data, neuroimaging data, and clinical information with machine learning and artificial intelligence algorithms can lead to better disease subtyping, personalized treatment approaches, and predictive modeling for CFS/ME [26]. Lastly, wearable devices and digital health technologies can offer valuable real-time data on physiological parameters, activity levels, and sleep patterns, contributing to a better understanding of CFS/ME symptom fluctuations and management [42].

The use of these advanced technologies has the potential to significantly influence future CFS/ME research and clinical management. Genomic studies, such as GWAS and transcriptomic analyses, can help identify genetic markers associated with CFS/ME, paving the way for more personalized treatment approaches based on a patient's unique genetic profile. Metabolomics and proteomics analyses can uncover specific metabolic and protein expression abnormalities in CFS/ME, possibly leading to the identification of reliable biomarkers that can improve diagnosis and track disease progression.

Advanced neuroimaging techniques, including fMRI and DTI, provide insights into brain structure and function in CFS/ME patients, helping identify potential neurological abnormalities [24]. This information could guide the development of targeted therapeutic strategies aimed at neurological symptoms, such as cognitive dysfunction. Integrating large-scale omics data with artificial intelligence (AI) and machine learning algorithms may lead to better disease subtyping and predictive modeling, which could inform individualized treatment plans [49]. Additionally, wearable devices and digital health technologies could gather real-time data on patients' physiological parameters and activity levels, offering a dynamic means to monitor symptom fluctuations and potentially optimize ongoing management strategies. Together, these technologies have the capacity to revolutionize our understanding of CFS/ME, guiding more effective and targeted interventions.

Detection of Chronic Fatigue Through Machine Learning Algorithms

Understanding fatigue involves understanding multiple biological, physiological, behavioral, and philosophical data points. Fatigue research involves using magnetic resonance imaging techniques, recording human physiological and behavioral data points, and using this collective data to train and test machine learning algorithms. A study in 2018 showed how machine learning algorithms can help in fatigue research [49]. They used structural MRI, classified them from healthy human controls, and compared them with machine learning-based classification based on patient self-reporting [49]. They found that morphological classification using MRI helps screen chronic fatigue patients; however, self-reporting methods or questionnaires incorporated with machine learning algorithms show early prediction and early intervention opportunities [49-51].

## Conclusions

Diagnosing and treating CFS/ME is complex due to its overlapping symptoms with other conditions and the absence of specific biomarkers. Abnormal immune function, neuroinflammation, and dysregulation of the hypothalamic-pituitary-adrenal (HPA) axis (regulating stress responses through hormone secretion, particularly cortisol) suggest potential mechanisms underlying CFS/ME. The Fukuda and International Consensus Criteria offer guidance, but more research is needed to better understand the pathophysiology, etiology, and treatment options. Non-pharmacological therapies, such as CBT and pacing strategies, show varying efficacy and are recommended based on individual patient tolerance. However, therapies such as GET remain controversial due to reports of symptom exacerbation, indicating the need for more patient-centered research. This highlights the importance of accurate diagnosis and further studies into genetic variables, biomarkers, and personalized treatment strategies. Advancements in genomics and neuroimaging hold promise for improving patient outcomes by enhancing our understanding of the disease.
